# Development of Multiple Cross Displacement Amplification Label-Based Gold Nanoparticles Lateral Flow Biosensor for Detection of *Shigella* spp.

**DOI:** 10.3389/fmicb.2016.01834

**Published:** 2016-11-18

**Authors:** Yi Wang, Yan Wang, Jianguo Xu, Changyun Ye

**Affiliations:** State Key Laboratory of Infectious Disease Prevention and Control, National Institute for Communicable Disease Control and Prevention, Collaborative Innovation Center for Diagnosis and Treatment of Infectious Diseases, Chinese Center for Disease Control and PreventionBeijing, China

**Keywords:** *Shigella* spp., multiple cross displacement amplification, lateral flow biosensor, MCDA-LFB, limit of detection

## Abstract

*Shigella* spp., the etiological agent of shigellosis or “bacillary dysentery,” are responsible for considerable morbidity and mortality in excess of a million deaths globally per year. Although PCR-based techniques (such as PCR-based dipstick biosensors) have been used for the molecular diagnosis of infectious disease, these assays were restricted due to the need for a sophisticated thermal cycling apparatus to denature target templates. To facilitate simple and rapid detection of target pathogens, we successfully devised an inexpensive, reliable and nearly instrument-free molecular technique, which incorporates multiple cross displacement amplification (MCDA) combined with a newly designed lateral flow biosensor (LFB) for visual, sensitive and specific detection of *Shigella*. The MCDA-LFB assay was conducted at 65°C for only 20 min during the amplification stage, and then products were directly analyzed on the biosensor, alleviating the use of special reagents, electrophoresis equipment and amplicon detection instruments. The entire process, including specimen processing (35 min), amplification (20) and detection (2–5 min), can be finished within 1 h. The MCDA-LFB assay demonstrated high specificity for *Shigella* detection. The analytical sensitivity of the assay was 10 fg of genomic templates per reaction in pure culture and 5.86 CFU per tube in human fecal samples, which was consistent with MCDA by colorimetric indicator, gel electrophoresis, real time turbidity and fluorescence detection. Hence, the simplicity, rapidity and nearly instrument-free platform of the MCDA-LFB assay make it practical for ‘on-site’ diagnosis, point-of-care testing and more. Moreover, the proof-of-concept approach can be reconfigured to detect a wide variety of target sequences by re-designing the specific MCDA primers.

## Introduction

*Shigella* spp. are exquisitely fastidious gram-negative pathogens that are responsible for as many as 167 million cases of shigellosis worldwide, resulting in a million deaths annually (Schroeder and Hilbi, 2008). Four *Shigella* species, including *S. sonnei, S. boydii, S. flexneri*, and *S. dysenteriae*, are considered as pathogenic to humans, particularly in young children (Koh et al., 2012). The typical symptoms of *Shigella* infection include dysentery and/or diarrhea with frequent mucoid boldly stools, fever, abdominal pain, tenesmus and malaise (Khan et al., 2013). The individuals, including young children, older adults and immune-compromised populations, may be at more risk for *Shigella* infection (Njuguna et al., 2013). The low infective dose (10 cells) of *Shigella* permits the disease to be effectively spread by contaminated food or water, and also by person-to-person contact, thus the foodborne or waterborne outbreaks of *Shigella* are common (Haley et al., 2010; Nygren et al., 2013; Baker et al., 2015). Herein, a reliable detection tool is needed to offer accurate diagnosis of *Shigella* to achieve infection control, clinical care and epidemiologic investigations.

The traditional detection of *Shigella* relies on culture-based methods, while only a small fraction of the actual shigellosis cases can be identified (Echeverria et al., 1991). Moreover, the growth, and thus the identification of these pathogens is frequently further impaired by ongoing antimicrobial therapy prior to specimen collection. The molecular detection techniques, such as PCR-based protocols, which overcome some of disadvantages posed by culture methods, are employed for the diagnostic of *Shigella* spp. (McKillip and Drake, 2004; Warren et al., 2006; Mandal et al., 2011; Villalobo and Torres, 1998). These methodologies require a sophisticated thermal cycling apparatus to denature target templates and still analysis of the amplified products with either agarose gel electrophoresis or probe hybridization techniques, which significantly hampered its application in the laboratories with limited resources settings (Wang et al., 2015a,b, 2016a). Although, newer approaches, including chemical and biological sensors, have been reported to be very rapid, sensitive and specific for detecting PCR amplicons of different target, thermal cycling of PCR-based methods during the amplification stage imposed instrumental constraints, limiting these assays to a low-resource setting (Chua et al., 2011; Liao et al., 2016). As such, the suitable detection assays using a simple, rapid, sensitive and specific technique are continuously required for the effective control and prevention of *Shigella*.

The growing use of molecular diagnostic methods has emphasized speed, simplicity and inexpensiveness as key criteria for adoption in ‘on-site’ analysis, field diagnosis and point-of-care testing and more, and the isothermal amplification technologies were well-suited for these application. Among dozens of isothermal nucleic acid amplification technologies, a few of these techniques (e.g., RCA, rolling circle amplification; LAMP, loop-mediated isothermal amplification; CPA, cross priming amplification) can efficiently achieve amplification using only one enzyme (Zhao et al., 2015). However, RCA was limited to amplify the circular target DNA, and a ligation process before amplification was always conducted for the specific recognition of a sequence. Although LAMP and CPA assays displayed high amplification efficiency comparable to that of the PCR method, the marginal amounts of nucleic acid sequences were still difficultly to analyze in various samples (Wang et al., 2016b).

More recently, multiple cross displacement amplification (MCDA) (Chinese IP Office Patent Application CN201510280765.X) was successfully established to overcome the technical barriers posed by current isothermal amplification strategies, and the mechanism and rationale of MCDA technique have been described in details (Wang et al., 2015c). MCDA has exhibited unique advantages of simplicity, rapidity, sensitivity, specificity and repeatability, generating amplicons from as few as three bacterial cells. The gold nanoparticle-based immunochromatographic technique is another strategy that has been widely used for the detection of amplicons yielded by various nucleic acid amplification-based assays (Vikesland and Wigginton, 2010). Here, the amplicon detection using gold nanoparticle-based dipstick biosensor was employed to simplify and accelerate the process of interpreting MCDA approach results. In the current report, we devised a MCDA assay combined with lateral flow biosensor (MCDA-LFB) for simple, rapid, sensitive and accurate visual detection of target sequence. As a proof of concept, *Shigella* was detected by MCDA-LFB assay to demonstrate the capability of target analysis. The performance of the MCDA-LFB methodology in detecting *Shigella* from pure culture and practical sample was successfully evaluated.

## Materials and Methods

### Reagents and Instruments

The sample pad, conjugate pad, nitrocellulose membrane (NC), absorbent pad and backing card were purchased from the Jie Yi Biotechnology Co., Ltd. (Shanghai, China). The streptavidin-immobilized gold nanoparticles (SA-G), rabbit anti-fluorescein antibody (anti-FITC) and biotinylated bovine serum albumin (biotin-BSA) were purchased from the Resenbio Co., Ltd. (XiAn, China). The QIAamp DNA Stool Mini Kit and QIAamp DNA Mini Kit (QIAamp DNA minikits; Qiagen, Hilden, Germany) were purchased from Qiagen (Beijing, China). Loopamp^TM^ Fluorescent Detection Reagent (FD) and the Loopamp kits were purchased from Eiken Chemical (Beijing, China).

### Preparation of Gold Nanoparticle-Based Dipstick Biosensor

**FIGURE 1:**
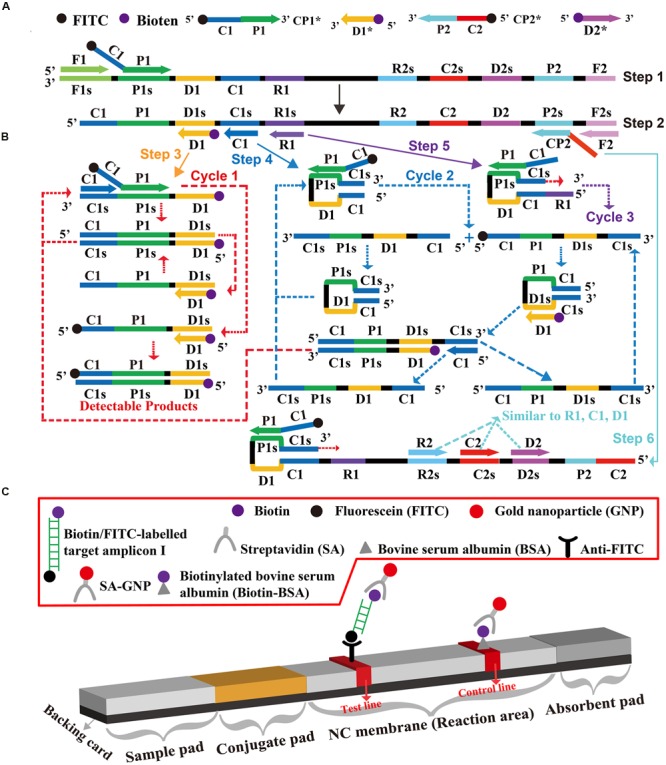
**The outline of multiple cross displacement amplification combined with lateral flow biosensor. (A)** Schematic depiction of the new cross primer (CP1^∗^) and amplification primer (D1^∗^). **(B)** Outline of multiple cross displacement amplification with CP1^∗^ and D1^∗^. **(C)** Schematic illustration of the principle of lateral flow biosensor for visualization of MCDA amplicons.

The dry-reagent dipstick (5 mm × 70 mm), illustrated in **Figure 1**, consisted of an absorbent pad, a NC membrane, a conjugate pad and an immersion pad assembled on a plastic adhesive backing card. The capture reagents, including anti-FITC (0.15 mg/ml) and biotin-BSA (4 mg/ml) in 0.01 M phosphate-buffered saline (PBS, PH 7.4), were dispensed onto the reaction regions. On the NC membrane, there are two zones as the test zone (conjugated with anti-FITC) and control zone (conjugated with biotin-BSA), with each line separated by 5 mm. SA-G in 0.01M PBS (PH 7.4) was deposited on the conjugate pad of the biosensor. Then, the assembled cards were cut at 5 mm widths, and the biosensors were dryly stored at the room temperature until use.

### Visual Detection of MCDA Products Using the Biosensor

A 0.5 μl aliquot of MCDA amplicons was deposited to the sample application area of the biosensor. Then, the strip was directly immersed into 120 μl of running buffer (10 mM PBS, PH 7.4 with 1% Tween 20) and the biosensor allowed absorbing the whole running buffer. After 2 min, the MCDA product detection was visualized in the form of red lines on the NC membrane.

### Primer Design for MCDA Approach

**FIGURE 2:**
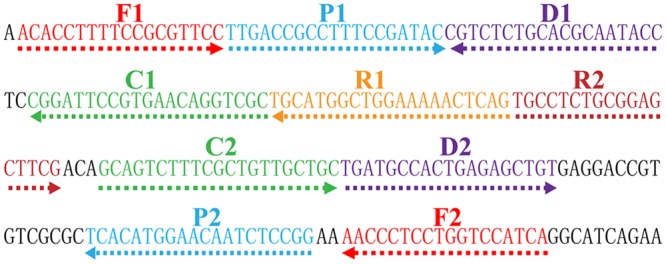
**Location and sequence of *ipaH* gene (*Shigella* app.-specific gene) used to design multiple cross displacement amplification primers.** The nucleotide sequence of the sense strand of *ipaH* was exhibited. Right arrows and left arrows indicate sense and complementary sequences that were used.

In order to design *Shigella* spp. specific MCDA primers, the nucleotide sequence of the specific *ipaH* gene (GenBank accession no. M32063) was downloaded from the NCBI Genbank database, and a set of MCDA primers was designed by PrimerExplorer V4 (Eiken Chemical, Japan) and primer software PRIMER PREMIER 5.0 (Thiem et al., 2004). Blast analysis demonstrated that the MCDA primer set was specific for *Shigella* spp. strains. The details of primer design, primers sequences, locations and modifications of MCDA primers were displayed in **Figure 2** and **Table 1**. All of the oligomers were synthesized and purified by TsingKe Biological Technology (Beijing, China) at HPLC purification grade.

**Table 1:** The primers used in this study.

Primers name^a^	Sequences and modifications^b^	Length^c^	Gene
Shi-F1	5′-ACACCTTTTCCGCGTTCC-3′	18 nt	*ipaH*
Shi-F2	5′-TGATGGACCAGGAGGGTT-3′	18 nt	
Shi-CP1	5′-GCGACCTGTTCACGGAATCCG-TTGACCGCCTTTCCGATAC-3′	40 mer	
Shi-CP1^∗^	5′-FITC-GCGACCTGTTCACGGAATCCG-TTGACCGCCTTTCCGATAC-3′	40 mer	
Shi-E-CP1	5′-Hex-TGCAATG-GCGACCT(BHQ1)GTTCACGGAATCCG-TTGACCGCCTTTCCGATAC-3′	47 mer	
Shi-CP2	5′-GCAGTCTTTCGCTGTTGCTGC-CCGGAGATTGTTCCATGTGA-3′	41 mer	
Shi-C1	5′-GCGACCTGTTCACGGAATCCG-3′	21 nt	
Shi-C2	5′-GCAGTCTTTCGCTGTTGCTGC-3′	21 nt	
Shi-D1	5′-GGTATTGCGTGCAGAGACG-3′	19 nt	
Shi-D1^∗^	5′-Biotin-GGTATTGCGTGCAGAGACG-3	19 nt	
Shi-D2	5′-TGATGCCACTGAGAGCTGT-3′	19 nt	
Shi-R1	5′-CTGAGTTTTTCCAGCCATGCA-3′	21 nt	
Shi-R2	5′-TGCCTCTGCGGAGCTTCG-3′	18 nt	

*^a^Shi, *shigella*; Shi-CP1^∗^, 5′-labeled with FITC when used in MCDA-LFB assay; Shi-D1^∗^, 5′-labeled with biotin when used in MCDA-LFB assay; Shi-E-CP1, 5′-labeled with Hex when used in ET-MCDA assay; ^b^Hex, hexachloro-fluorescein; FITC, fluorescein isothiocyanate. ^c^mer, monomeric unit; nt, nucleitide.*

### Bacterial Strains and Genomic Template Preparation

A total of 60 bacterial strains were used in this study (**Table 2**). Twenty-three *Shigella* strains and 37 non-*Shigella* strains were included to test the specificity of the MCDA-LFB assay. All bacterial strains were stored in 10% (w/v) glycerol broth at -70°C and then were refreshed three times on nutrient agar plate at 37°C. The genomic DNA templates were extracted from all culture strains using DNA extraction kits according to the manufacturer’s instructions. The extracted templates were examined with ultraviolet spectrophotometer (Nano drop ND-1000, Calibre, Beijing, China) at A260/280 and stored under at -20°C before the templates were used. The strains of *S. flexneri* serovar 1d (ICDC-NPS001) were applied for confirmation performance, optimal temperature, sensitivity analysis and practical application conducted in the report. Moreover, the genomic templates of *S. flexneri* serovar 1d (ICDC-NPS001) were serially diluted (10 ng, 10 pg, 10 fg, 1 fg, and 0.1 fg) for sensitivity evaluation of MCDA-LFB detection.

**Table 2:** Bacterial strains used in this study.

Bacteria	Serovar/Species	Strain no. (source of strain)^a^	No. of strains
*Shigella flexneri*	1d	ICDC-NPS001	1
	4a	ICDC-NPS002	1
	5a	ICDC-NPS003	1
	2b	ICDC-NPS004	1
	1b	ICDC-NPS005	1
	3a	ICDC-NPS006	1
	4av	ICDC-NPS007	1
	3b	ICDC-NPS008	1
	5b	ICDC-NPS009	1
	Y	ICDC-NPS010	1
	Yv	ICDC-NPS011	1
	1a	ICDC-NPS012	1
	X	ICDC-NPS013	1
	Xv	ICDC-NPS014	1
	F6	ICDC-NPS015	1
	7b	ICDC-NPS016	1
	2a_1_	ICDC-NPS017	1
	4b	ICDC-NPS018	1
*Shigella boydii*	U	Isolated strains (ICDC)	1
*Shigella dysenteriae*	U	Isolated strains (ICDC)	2
*Shigella sonneri*	U	Isolated strains (ICDC)	2
*Salmonella*	Choleraesuis	ICDC-NPSa001	1
	U	Isolated strains (ICDC)	10
*Listeria seeligeri*	U	ATCC35967	1
*Listeria grayii*	U	Isolated strains (ICDC)	1
*Listeria monocytogenes*	4a	ATCC19114	1
*Listeria welshimeri*	U	ATCC35897	1
*Listeria ivanovii*	U	Isolated strains (ICDC)	1
*Bacillus cereus*	U	Isolated strains (ICDC)	1
*Enteropathogenic Escherichia coli*	U	Isolated strains (ICDC)	1
*Enterotoxigenic Escherichia coli*	U	Isolated strains (ICDC)	1
*Enteroaggregative Escherichia coli*	U	Isolated strains (ICDC)	1
*Enteroinvasive Escherichia coli*	U	Isolated strains (ICDC)	1
*Enterohemorrhagic Escherichia coli*	U	EDL933	1
*Plesiomonas shigelloides*	U	ATCC51903	1
*Campylobacter jejuni*	U	ATCC33291	1
*Enterobacter cloacae*	U	Isolated strains (ICDC)	1
*Enterococcus faecalis*	U	ATCC35667	1
*Enterococcus faecium*	U	Isolated strains (ICDC)	
*Yersinia enterocolitica*	U	ATCC23715	1
*Streptococcus pneumoniae*	U	Isolated strains (ICDC)	1
*Aeromonas hydrophila*	U	ATCC7966	1
*Vibrio vulnificus*	U	Isolated strains (ICDC)	1
*Proteus vulgaris*	U	Isolated strains (ICDC)	1
*Vibrio fluvialis*	U	Isolated strains (ICDC)	1
*Streptococcus bovis*	U	Isolated strains (ICDC)	1
*Vibrio parahaemolyticus*	U	ATCC17802	1
*Klebsiella pneumoniae*	U	ATCC700603	1
*Bntorobater sakazakii*	U	Isolated strains (ICDC)	1

***^a^**U, unidentified serotype; ATCC, American Type Culture Collection; ICDC, National Institute for Communicable Disease Control and Prevention, Chinese Center for Disease Control and Prevention.*

### The Standard MCDA Assay

In order to assess the feasibility of *ipaH*-MCDA primers, the MCDA reaction was conducted as the standard MCDA condition, which has been reported in previous report (Wang et al., 2015c). In brief, the MCDA assay was carried out in 25-μl amplification mixtures containing the following components: 0.4 μM each of displacement primers F1 and F2, 0.8 μM each of amplification primers C1 and C2, 1.2 μM each of amplification primers R1, R2, D1^∗^ and D2, 1.2 μM each of cross primers CP1^∗^ and CP1, 2.4 μM cross primer CP2, 12.5 μl 2× reaction mix (Loopamp kits), 1.25 μl of *Bst* DNA polymerase (10 U) and 1 μl DNA template. Four monitoring techniques, including colorimetric indicator (FD), gel electrophoresis, turbidimeters (LA-320C) and LFB detection, were employed to analyze the MCDA products. Furthermore, the endonuclease restriction-mediated real-time multiple cross displacement amplification (ET-MCDA), which was reported in a recent study, was employed to achieve real time fluorescence measurement of MCDA reaction (Wang et al., 2015c, 2016b).

Then, we tested the optimal reaction temperature of *ipaH*-MCDA primers. The MCDA reaction mixtures were performed at a constant temperature ranging from 60°C to 67°C for 1 h and then incubated at 85°C for 5 min to stop the amplification. Mixtures with 1 μl genomic template of *Listeria monocytogens* strain (*L. monocytogenes*, ATCC19114) and *Salmonella* strain (ICDC-NPsa001) were used as negative controls, and mixtures with 1 μl double distilled water (DW) were used as a blank control.

### The Analytical Sensitivity of the *Shigella*-MCDA by Five Monitoring Techniques

The templates of *S. flexneri* serovar 1d (ICDC-NPS001) were serially diluted to confirm the limit of detection (LoD), which was defined by genomic DNA amount of the template. The analytical sensitivity of MCDA by colorimetric indicator (FD reagent), real time turbidity, 2% agarose gel electrophoresis, real time fluorescence and LFB detection was determined as described above. At least three replicates of each dilution were examined to test the analytical sensitivity.

### The Analytical Specificity of the MCDA-LFB Approach

In order to assess the analytical specificity of MCDA-LFB methodology, the MCDA reactions were carried out under the conditions described above with purely genomic templates from 23 *Shigella* strains and 37 non-*Shigella* strains (**Table 2**). The MCDA products were tested using 2.5% agarose gel electrophoresis and LFB detection. Analysis of each sample was examined in at least two independent experiments.

### Examination of MCDA-LFB Assay Using Simulated Human Fecal Specimens

Human fecal samples were acquired from a healthy donor with the written informed consent. Our study was reviewed and approved by the ethics committee of the National Institute for Communicable Disease Control and Prevention, China CDC, according to the medical research regulations of the Ministry of Health China (Approval No. ICDC2014003).

In order to evaluate the suitability of MCDA-LFB technique as a surveillance tool for *Shigella*, the MCDA-LFB assay was applied to rapidly diagnose the target pathogens in human fecal samples. Firstly, the human fecal samples were confirmed as being *Shigella*-negative by culture-based methods and PCR detection. Then, to test the minimal detectable colony forming units (CFUs), the cultures with *S. flexneri* strains were serially diluted (10^-1^ to 10^-9^), and the aliquots of 100 μl appropriate dilution (10^-6^) was spread in triplicate onto brain heart infusion (BHI) agar. The CFUs were counted after 24 h at 37°C. Simultaneously, the aliquots of 100 μl appropriate dilution (10^-3^ to 10^-8^) with *S. flexneri* strains were inoculated into the fecal samples (0.2 g), and the number of *Shigella* was adjusted to approximate 1.42 × 10^6^, 1.42 × 10^5^, 1.42 × 10^4^, 1.42 × 10^3^, 1.42 × 10^2^ and 1.41 × 10^1^ CFU/g. Then, the artificially contaminated stool samples were applied to extract the genomic DNA templates, and the supernatants (2 μl) were used for MCDA detections. Non-contaminated fecal sample was used as negative control and this analysis was independently conducted in triplicate. The MCDA products were also analyzed by colorimetric indicator (FD reagent), real time turbidity, 2% agarose gel electrophoresis, real time fluorescence and LFB detection as described above.

## Results

### Development of the MCDA-LFB Assay

A schematic of MCDA-LFB technique was shown in **Figure 1**. In the MCDA-LFB system, the cross primer (CP1 or CP2) involved in MCDA reaction were labeled at the 5′ end with FITC, and the amplification primers (D1 or D2) were modified at the 5′ end with biotin (**Figure 1A**). The new CP1, CP2, D1, and D2 primers were named as CP1^∗^, CP2^∗^, D1^∗^, and D2^∗^, respectively. For clarity, the CP2^∗^ and D2^∗^ primers were not displayed in outline of MCDA reaction during the reaction stage (**Figure 1B**). The CP1^∗^ primer initiated MCDA reaction at the P1s site of the target sequence, and the newly synthesized strand was displaced by upstream synthesis from the primer F1 (Step 1). Five primers (D1^∗^, C1, R1, CP2, and F2) annealed to the newly generated strand, and then the Bst polymerase extended in tandem producing four different products (Step 2). The D1^∗^ product was used as the template by C1 and CP1^∗^ primers, enter a cyclic process (Step 3, Cycle 1). In the cycle, a larger amounts of double-labled detectable amplicons, which contained biotin-labeled D1^∗^ primer and a FITC-labeled CP1^∗^ primer, were successfully yielded. The details of the reaction process for C1, R1, and CP2 products (Step 4, 5, 6) has been reported in previous study (Wang et al., 2015c). In addition, a double-labeled detectable product (CP2^∗^/D2^∗^ product), which was similar to the detectable CP1^∗^/D1^∗^ product, could be formed when the CP2 primer was modified with a FITC at the 5′ end and D2 primer for biotin.

The principle of LFB for visualization of MCDA amplicons was exhibited in **Figure 1C**. The LFB detected MCDA amplicons through specific recognition of the FITC labels at the end of products, which were formed by using FITC labeled primers (CP1^∗^ primer). The other end, the amplicons labeled with biotin binded streptavidin-conjugated gold nanoparticles for visualization. The MCDA products were deposited onto on the sample application region of the biosensor, and then the biosensor was directly immersed in the running buffer. The running buffer moved along the biosensor by capillary action, which rehydrated the immobilized detector reagents (SA-G). The target amplicons was specifically captured by the immobilized anti-FITC at the first test zone and detector reagents rapidly accumulate in the reaction zone of the strip through biotion/streptavidin interaction, resulting in a visual red colored line on the test region. The proper function of the strip is demonstrated by the control line formation which contained biotinylated bovine serum albumin that captured excess detector reagent.

### Confirmation and Detection *Shigella*-MCDA-LFB Products

**FIGURE 3:**
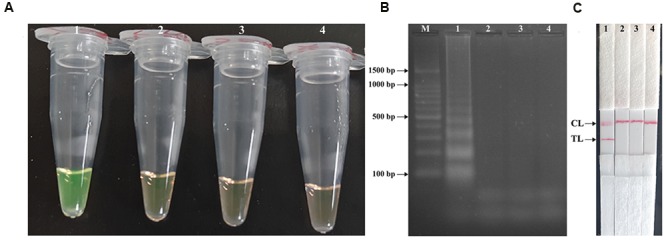
**Detection and confirmation of *Shigella*-MCDA products. (A)** Amplification products of *Shigella*-MCDA assay were visually detected by observation of the color change: tube 1, positive amplification of *Shigella flexneri* strain (ICDC-NPS001); negative control of *Listeria monocytogenes* strain (ATCC19114); negative control of *Salmonella* strain (ICDC-NPSa001); blank control (DW). **(B)** Agarose gel electrophoresis of *Shigella*-MCDA products was shown: lane M, DNA marker DL100; lane 1, *Shigella*-MCDA products of *Shigella flexneris* (ICDC-NPS001); lane 2, negative control (*Listeria monocytogenes*, ATCC19114); negative control (*Salmonella*, ICDC-NPSa001); lane 4, blank control (DW); **(C)** Lateral flow biosensor applied for visual detection of *Shigella* MCDA products: strip 1, positive amplification of *Shigella flexneris* (ICDC-NPS001); strip 2, negative control (*Listeria monocytogenes*, ATCC19114); strip 3, negative control (*Salmonella*, ICDC-NPSa001); strip 4, blank control (DW).

In order to verify the feasibility of *Shigell*-MCDA primers, the MCDA reactions were carried out in the presence or absence of genomic DNA templates within 60 min at a constant temperature (65°C). Three monitoring techniques, including colorimetric indicator (FD reagent), gel electrophoresis analysis and LFB detection, were employed to confirm the *Shigella*-MCDA products. A color shift of positive amplification in *Shigella*-MCDA tubes was directly observed from light gray to green (**Figure 3A**). The positive MCDA products were seen many bands of different sizes in a typical ladder-like pattern on ethidium bromide-stained 2% agarose gel electrophoresis, but not in the negative and blank control (**Figure 3B**). It was also observed that two visible red bands (Test line, TL; Control line, CL) were seen in positive amplifications, and only the CL were seen in negative and blank controls (**Figure 3C**). Therefore, the MCDA primer set was a good candidate for establishment of the MCDA-LFB method for *Shigella* detection.

### The Optimal Amplification Temperature of the MCDA-LFB Assay

**FIGURE 4:**
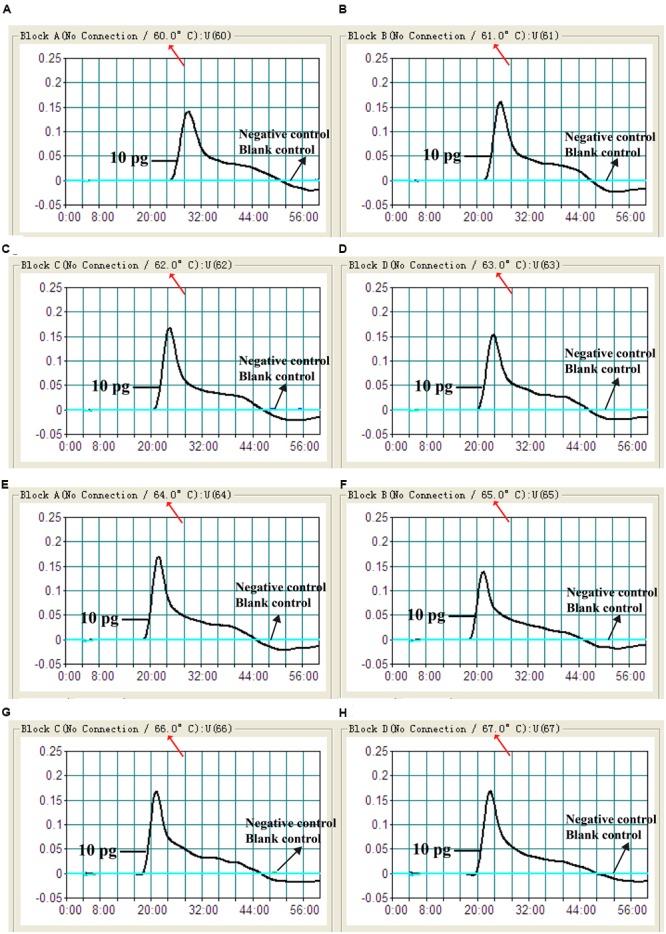
**The optimal amplification temperature for *Shigella*-MCDA primer sets.** The standard MCDA reactions for detection of *Shigella* were monitored by real-time measurement of turbidity and the corresponding curves of concentrations of DNA were marked in the figures. The threshold value was 0.1 and the turbidity of >0.1 was considered to be positive. Eight kinetic graphs **(A–H)** were generated at various temperatures (60–67°C, 1°C intervals) with target pathogens DNA at the level of 10 pg per reaction. The graphs from **(D–H)** showed robust amplification.

In order to examine the optimal assay temperature during the amplification stage, the *Shigella*-MCDA reactions were conducted at eight distinct temperatures (60°C–67°C) with 1°C intervals. The strain *S. flexneri* serovar 1d (ICDC-NPS001) was employed as the positive control to evaluate the optimal amplification temperature at the level of 10 pg genomic templates per reaction. The reactions were analyzed by means of real time turbidity detection and the typical kinetics graphs corresponding to eight temperatures were obtained (**Figure 4**). Eight reaction temperatures provided a robust signal, with the faster amplifications generated from assay temperature of 63°C–67°C, which were recommended as the standard temperature for *Shigella*-MCDA-LFB assay during the amplification stage. The assay temperature of 65°C was used for the rest of MCDA-LFB tests conducted in this study.

### Analytical Sensitivity of MCDA-LFB Technique in Pure Culture

**FIGURE 5:**
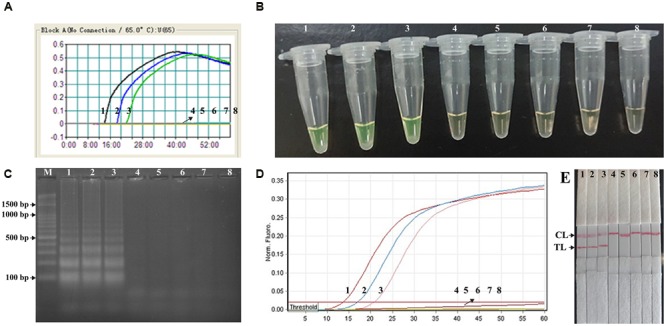
**Analytical sensitivity of *Shigella*-MCDA assay using serially diluted genomic DNA with *Shigella flexneris* strain ICDC-NPs001.** Five monitoring techniques, including real time turbidity **(A)**, colorimetric indicator (FD) **(B)**, gel electrophoresis **(C)**, real time fluorescence **(D)** and lateral flow biosensor **(E)**, were applied for analyzing the amplification products. The serial dilutions (10 ng, 10 pg, 10 fg, 1 fg, and 0.1 fg) of target templates were subjected to standard MCDA or ET-MCDA reactions. Turbidity signals **(A)**/Tubes **(B)**/Lanes **(C)**, Fluorescence signals **(D)**/Strips **(E)** 1–8 represented the DNA levels of 10 ng, 10 pg, 10 fg, 1 fg, and 0.1 fg per reaction, negative control (10 pg of *Listeria monocytogenes* genomic DNA), negative control (10 pg of *Salmonella* genomic DNA) and blank control (DW). The genomic DNA levels of 10 ng, 10 pg, and 10 fg per reaction produced the positive reactions.

The analytical sensitivity of MCDA-LFB technique on *Shigella* was determined by analyzing the products yielded from the serial dilutions (10 ng, 10 pg, 10 fg, 1 fg, and 0.1 fg per microliter) of *Shigella* genomic DNA in triplicate (**Figure 5**). The *Shigell*-MCDA reactions were monitored by real time measurement of turbidity and the LoD of MCDA-LFB assay for *Shigella* detection was 10 fg of genomic templates per reaction (**Figure 5A**). By the FD reagent, a color shift of positive amplification in *Shigella*-MCDA tubes was directly observed from light gray to green (**Figure 5B**). Then, the *Shigella*-MCDA products were analyzed by 2% agarose gel electrophoresis and positive products were observed as the ladder-like patterns but not in negative reactions, negative control and blank control (**Figure 5C**). The biosensor was also subjected to detect the *Shigella*-MCDA products (**Figure 5E**). As expected, the biosensor exhibited clear visible red bands for both TL and CL when the products came from positive MCDA amplifications, and only the CL were generated from for negative MCDA amplifications, negative control and blank control. The LoD of MCDA-LFB assay for detecting *ipaH* gene was also 10 fg of genomic templates per reaction. Moreover, the LoD of ET-MCDA assay for *Shigella* detection was also 10 fg of genomic DNA in pure culture (**Figure 5D**). These results indicated that the analytical sensitivity by FD reagent, real time turbidity, real time fluorescence and agarose gel electrophoresis detection for *Shigella*-MCDA amplifications was conformity with biosensor analysis.

**FIGURE 6:**
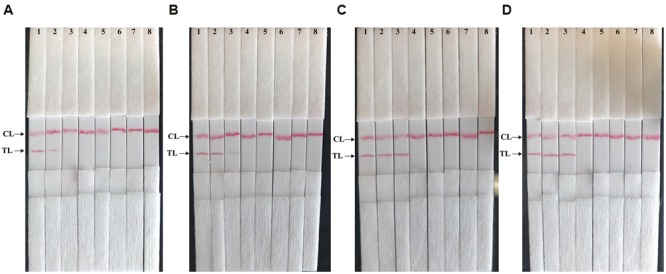
**The optimal duration of time required for MCDA-LFB assay.** Four different reaction times (**A**, 10 min; **B**, 15 min; **C**, 20 min; **D**, 25 min) were examined and compared at 65°C. Strips 1, 2, 3, 4, 5, 6, 7, and 8 represent DNA levels of 10 ng of *Shigella* templates, 10 pg of *Shigella* templates, 10 fg of *Shigella* templates, 1 fg of *Shigella* templates, 0.1 fg *Shigella* templates per tube, negative control (*L. monocytogenes*, 10 pg per reaction), negative control (*Salmonella*, 10 pg per reaction) and blank control (DW). The best sensitivity was seen when the amplification lasted for 20 min **(C)**.

Then, we assessed the optimal duration of time require for the MCDA-LFB assay during the amplification stage, and four different reaction times (10, 15, 20, and 25 min) were compared at 65°C according to the standard MCDA conditions. The lowest genomic DNA level (10 fg of *Shigella* templates per tube) showed two red bands when the reaction only lasted for 20 min at 65°C (**Figure 6**). A reaction time of 20 min was used as the optimal time for the MCDA-LFB assay during the reaction stage. Hence, the whole procedure, including specimen (such as fecal sample) processing (35 min), isothermal reaction (20 min), and result reporting (5 min), could be completed within 60 min.

### The Analytical Specificity of MCDA-LFB Assay

**FIGURE 7:**
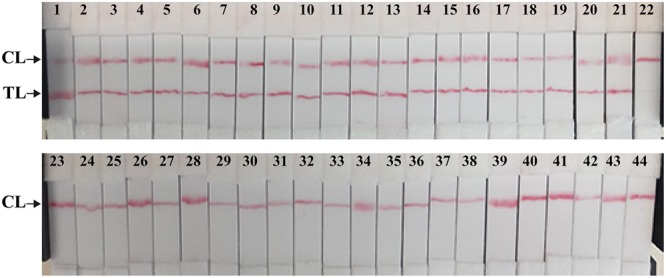
**The specificity of MCDA-LFB assay for different strains.** The MCDA reactions were conducted using different genomic DNA templates and were monitored by means of visual format. Biosensor 1–18, *Shigella flexneri* strains of serovar 1d (ICDC-NPS001), 4a (ICDC-NPS002), 5a (ICDC-NPS003), 2b (ICDC-NPS004), 1b (ICDC-NPS005), 3a (ICDC-NPS006), 4av (ICDC-NPS007), 3b (ICDC-NPS008), 5b (ICDC-NPS007), Y (ICDC-NPS010), Yv (ICDC-NPS011), 1a (ICDC-NPS012), X (ICDC-NPS013), Xv (ICDC-NPS014), F6 (ICDC-NPS015), 7b (ICDC-NPS016), 2a_1_ (ICDC-NPS017), 4b (ICDC-NPS018); biosensor 19–21, *Shigella boydii, Shigella sonneri* and *Shigella dysenteriae*; biosensor 22–43, *Enteropathogenic E. coli, Enterotoxigenic E. coli, Enteroaggregative E. coli, Enteroinvasive E. coli, Enterohemorrhagic E. coli, Plesiomonas shigelloides, Campylobacter jejuni, Enterobacter cloacae, Enterococcus faecalis, Enterococcus faecium, Yersinia enterocolitica, Streptococcus pneumonia, Aeromonas hydrophil, Vibrio vulnificus, Vibrio fluvialis, Vibrio parahaemolyticus, Klebsiella pneumonia, Bntorobater sakazakii, Bacillus cereus, Listeria grayii, Listeria welshimeri*, and *Listeria ivanovii*; biosensor 44, blank control (DW).

The analytical specificity of the MCDA-LFB technique was evaluated by MCDA-LFB amplification of genomic DNA extracted from 23 *Shigella* strains and 37 non-*Shigella* strains (roughly 10 ng of genomic templates for each pathogen). The detection was positive only for the four *Shigella* species, and was negative for non-*Shigella* species and blank control (**Figure 7**). As shown in **Figure 7**, two red bands, including TL and CL, appeared on the biosensor from the positive test, and only a red band at the control line appeared, indicating negative results for non-*Shigella* strains and blank control. The results demonstrated that the MCDA-LFB assay has a 100% analytical specificity for *Shigella* detection.

### MCDA-LFB Assay for Artificially Contaminated Fecal Samples

**FIGURE 8:**
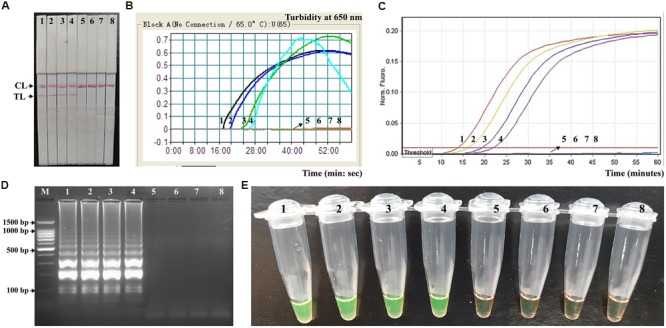
**Analytical sensitivity of MCDA-LFB for detecting target pathogens in artificially contaminated fecal samples.** Five monitoring techniques, including lateral flow biosensor **(A)**, real time turbidity **(B)**, real time fluorescence **(C)**, gel electrophoresis **(D)**, and colorimetric indicator (FD) **(E)**, were applied for analyzing the amplification products. The serial dilutions of target templates were subjected to standard MCDA or ET-MCDA reactions. Strips **(A)**/Turbidity signals **(B)**/Fluorescence signals **(C)**/Lanes **(D)**/Tubes **(E)** 1–8 represented the DNA levels of 5860 CFU, 586 CFU, 58.6 CFU, 5.86 CFU, 0.586 CFU and 0.0586 CFU per reaction, negative control (non-contaminated fecal sample) and blank control (DW). The genomic DNA levels of 5860 CFU, 586 CFU, 58.6 CFU and 5.86 CFU, per reaction produced the positive reactions.

In order to determine the suitability of the MCDA-LFB assay as a nucleic acid detection tool, the MCDA-LFB approach was examined by the artificially inoculating *Shigella* strains into human fecal samples. As shown in **Figure 8A**, the MCDA-LFB assay could generate positive results when the contaminated numbers of *Shigella* were more than 1.42 × 10^3^ CFU/g (∼5.68 CFU/reaction). The MCDA-LFB approach produced the negative results when the contaminated numbers of *Shigella* were lower than 1.42 × 10^2^ CFU/g (∼0.568 CFU/reaction). Only a red band at the control line appeared, indicating negative results for negative control and blank control. Thus, the LoD of MCDA-LFB method was 5.68 CFU per tube, which was consistent with MCDA-FD, MCDA-turbidity and MCDA-gel electrophoresis assays (**Figures 8B,D,E**). In contrast, the analytical sensitivity of ET-MCDA assay for detection of *Shigella* in fecal samples was also 5.68 CFU per reaction, which was as sensitive as MCDA-LFB detection (**Figures 8A,C**). The results indicated that the analytical sensitivity of MCDA-LFB assay was in complete accordance with MCDA-FD, MCDA-turbidity, MCDA-gel electrophoresis and ET-MCDA assays.

## Discussion

Species of the genus *Shigella* were the causative agents of shigellosis or “bacillary dysentery,” and responsible for 5–15% of all diarrheal episodes worldwide, disproportionately affecting children 5 years of age living in developing countries (Von Seidlein et al., 2006; Schroeder and Hilbi, 2008). Thus, a simple, rapid and accurate detection assay, which can be used in clinical laboratories, primary care facilities and resource-poor settings, is necessary. In this study, we successful developed a MCDA-LFB technique for simple, rapid, sensitive and specific detection of *Shigella* spp. as a valuable screening tool. Comparing with the currently existent PCR-based technologies, the MCDA-LFB assay during the reaction stage was preceded at a uniform temperature, alleviating the use of a sophisticated thermal cycling instrument, and only a water bath or heat block was need to conduct the reaction. Hence, the MCDA-LFB method developed here had the potential for point-of-care testing, field detection, ‘on-site’ diagnosis and more. Furthermore, only a reaction time of 20 min was required for the MCDA-LFB assay during the amplification stage. Consequently, the entire procedure, including specimen (such as stool sample) processing (35 min), isothermal reaction (20 min), and result reporting (5 min), could be completed within 60 min (**Figure 6**). The rapid detection of *Shigella* was valuable for determining the choice of treatment in clinical laboratories, especially in acute-care settings.

In the MCDA assay, CP1 and D1 primers, which involved in isothermal amplification, were labeled at the 5′ end with FITC and biotin, respectively (**Figure 1**). During the amplification stage, the double-labeled detectable amplicons were constructed, which were generated from FITC-labeled CP1 primers and biotin-labeled D1 primers. The end of the detectable products labeled with FITC could be captured by the anti-FITC body fixed on the first line of the biosensor, known as the test line. The other end of the amplicons labeled with biotin could bind streptavidin-conjugated gold nanoparticles for visualization. The excess streptavidin-conjugated color particles were captured by biotinylated bovine serum albumin located on the second line of strip, known as the control line, which validated the working condition of the biosensor. Importantly, the test results are displayed as colored bands visible by the naked eye about 2 min, thus the whole process of detection could be finished within 5 min.

In the MCDA-LFB system, the interpretation of test results is based on the appearance of red bands on the reaction pad. The presence of two red lines (TL and CL) on the biosensor indicated a positive result for *Shigella*, whereas only a red line appeared in the CL zone, indicating the negative result, negative control and blank control. Several other monitoring techniques, including colorimetric indicator (such as FD reagent), real time turbidity, gel electrophoresis and fluorescence detection, were employed to analyze the MCDA products. Firstly, the assessment of color shift with naked eye was potentially subjective, thus there was the possibility that a sample was somewhat ambiguous to the unaided eye when the concentration of target sequences was low. Secondly, due to use of ten primers, MCDA could produce a complex mixture of various amplicons, and thus these detection techniques (such as colorimetric indicator, real time turbidity and gel electrophoresis) could not distinguish the non-specific and specific products (Ge et al., 2013). Furthermore, these detection methods required a post detection procedure (gel electrophoresis), turbidimeter (real time turbidity detection), or a fluorescence instrument (real time fluorescence detection), and the resultant instrumental restraint could hamper the uptake of MCDA analysis in point-of use and field settings. In our report, the MCDA technique coupled a lateral flow strip offered a simple, rapid, cost-effective and nearly instrument-free platform for molecular testing with easily interpretable results. Moreover, the proof-of-concept method may be reconfigured to detect a wide variety of nucleic acid sequences by re-designing the specific MCDA primers.

The newly developed MCDA-LFB approach could detect as little as 10 fg of *Shigella* DNA per reaction in pure culture and 5.86 CFU per tube in human fecal samples, and the results were further confirmed by FD, real time turbidity, gel electrophoresis and real time fluorescence detection (**Figure 5** and **8**). The results showed the LFB technique was as sensitive as FD, real time turbidity, gel electrophoresis and real time florescence detection. Due to negate the need for special reagents, electrophoresis and amplificon detection equipment, the MCDA-LFB assay was more suitable than other MCDA-based methods for simple, rapid and specific detection in a variety of fields with short turnaround times. Moreover, the use of the ten specific primers targeting the *ipaH* gene (*Shigella* spp.-specific gene) provides a high degree of specificity for nucleic acid amplification, and the analytical specificity was successfully assessed in this study (**Figure 7**). The detection was positive only for the four *Shigella* species, and was negative for non-*Shigella* species and blank control. Hence, the MCDA-LFB assay offered a high degree of selectivity for detecting *Shigella*.

## Conclusion

A reliable MCDA-LFB technique was successfully devised for detection of *Shigella* app. causing severe diarrhea in both developed and developing countries, which could achieve the infection control, clinical care and epidemiologic investigations. The MCDA-LFB assay reported here is simple, sensitive and specific, and did not require special reagents and expensive apparatus. The use of the newly designed biosensor could offer a rapid, objective and easily interpretable readout of the assay’s results. Therefore, the *Shigella*-MCDA-LFB assay was especially useful in field, point-of-care and resource-limited settings. Furthermore, the proof-of-concept technique (MCDA-LFB) may be reconfigured to detect a wide variety of nucleic acid sequences by re-designing the specific MCDA primers.

## Author Contributions

Conceived and designed the experiments: YiW, JX and CY. Performed the experiments: YiW and YaW. Analyzed the data: YiW. Contributed reagents/materials/analysis tools: YiW, YaW, JX, and CY. Designed the software used in the analysis: YiW. Wrote the manuscript: YiW, JX, and CY.

## Disclosures

YW and CY have filed for a patent from the State Intellectual Property Office of the People’s Republic of China, which covers the novel assay and sequences included in this manuscript (Application number CN201610942289.8).

## Conflict of Interest Statement

The authors declare that the research was conducted in the absence of any commercial or financial relationships that could be construed as a potential conflict of interest.
